# Supplementary material for: In situ n-doped nanocrystalline electron-injection-layer for general-lighting quantum-dot LEDs
Source: Nat Commun. 2025 Apr 9;16:3362. doi: 10.1038/s41467-025-58471-5 (PMC11982379; doi:10.1038/s41467-025-58471-5)
Supplement: Supplementary file 1 — Supplementary Information [file 41467_2025_58471_MOESM1_ESM.pdf]

Supplementary information

In situ n-doped nanocrystalline electron-injection-layer for general-lighting quantum-dot LEDs

Authors: Yizhen Zheng[1]†, Xing Lin[2]†*, Jiongzhao Li[1], Jianan Chen[1], Wenhao Wu[1], Zixuan Song[2], Yuan Gao[3], Zhuang Hu[1], Huifeng Wang[1], Zikang Ye[1], Haiyan Qin[1], & Xiaogang Peng[1]*

Affiliations:

[1]Key Laboratory of Excited-State Materials of Zhejiang Province, Department of Chemistry, Zhejiang University, Hangzhou 310027, China.

[2]Key Laboratory of Excited-State Materials of Zhejiang Province, College of Information Science and Electronic Engineering, Zhejiang University, Hangzhou 310027, China.

[3]Najing Technology Corporation Ltd., Hangzhou 310027, China.

†These authors contributed equally to this work.

*Corresponding author. Email: lxing@zju.edu.cn (X.L.); xpeng@zju.edu.cn (X.P.)

This file includes:

Supplementary Notes 1-2

Supplementary Figures 1-23

Supplementary Tables 1

Supplementary Note 1. Comparison with other state-of-the-art techniques.

In the existing literature, high-performance QLEDs are predominantly encapsulated using resins that contain acidic components. However, these devices exhibit a notable positive aging effect. Related to this, the water-vapor (or acid-vapor) treatments without the metal electrodes were also explored[1], with barely any improvement in comparison with the devices with the acid-containing encapsulation. Jia et al have employed thiol ligands to modify ZnO[2], thereby mitigating luminance quenching of quantum dots and addressing charge imbalance. Despite these efforts, the quantum efficiency and luminance of the devices remain suboptimal. Chen et al discovered that incorporating an ultrathin buffer layer, composed of minute ZnO nanoparticles, between the emissive layer (EML) and the electron transport layer (ETL), could significantly bolster the shelf stability of the devices[1]. Chen and colleagues investigated the use of hydroxyl-stabilized SnO_2 as an electron transport layer (ETL) in QLEDs, with their luminance, external quantum efficiency (EQE), and operational lifetime falling short[3]. Chen[4] also applied ultraviolet (UV) irradiation to eliminate adsorbed oxygen and to augment the driving current, which did not result in an increase in the EQE of the devices.

Our reductive treatment approach effectively elevates the luminance and current density beyond the levels achieved with acid-containing resins, all while maintaining the quantum efficiency and operational lifetime.

Supplementary Note 2. Electro-chemical reduction occurs during water-vapor and oxygen treatment.

The impact of water-vapor treatment on QLEDs with inert metal electrodes, such as silver and gold, is subtly distinct from that observed with active metals. Initially, QLEDs with a silver electrode exhibit poor performance post-treatment, which improves with successive *J-V* sweeps, as documented in Supplementary Fig. 21d-f for silver and g-i for gold. Ultimately, the electron conductivity and hole blocking of these devices align with those of standard QLEDs with aluminum electrodes.

Take the one with silver top electrode as an example. During the 1st *J-V* sweep below ~2.6 V, the current density (high hole leakage current and decent luminescence current), luminance (much higher brightness than the pristine devices), and EQE (similar to the pristine devices) are very similar to the QLEDs with the water-treatment prior to the deposition of a top electrode (data not shown). After 6 cycles of *J-V* sweeps (up to 4 V), the hole leakage current is gradually suppressed (in the voltage range above 1.8 V), luminance increases to the higher and higher level, and the maximum EQE finally researches >20%. These results imply that, under a significant bias, active reductive species (e.g., hydrogen radicals) are generated through electrochemical reactions of water on the Ag nanostructures, which eliminates the strongly adsorbed oxidative species (deep traps) to improve both electron conductivity and hole blockage of the ZnMgO layer.

This enhancement is akin to the electrochemical reduction of zinc-carboxylate or cadmium-carboxylate ligands in QLEDs[5], and is likely due to the generation of active hydrogen radicals on the nanostructures of silver or gold through the electrochemical reduction of water[6]. Notably, excessive water-vapor treatment leads to the formation of small bubbles in QLEDs with gold electrodes after *J-V* sweeps, indicating the production of hydrogen gas in this electrochemical process.

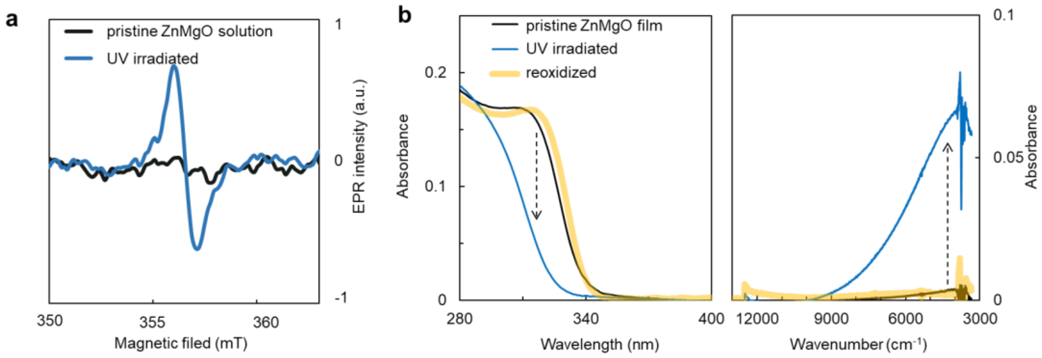

Supplementary Figure 1. n-doping of ZnMgO with UV irradiation

(a) The 298K EPR spectra of pristine (black) and UV-irradiated (blue) ZnMgO nanocrystals (in ethanol). (b) The ultraviolet-visible-near-infrared absorption spectra of pristine (black), UV-irradiated (blue) and reoxidized (yellow) ZnMgO films.

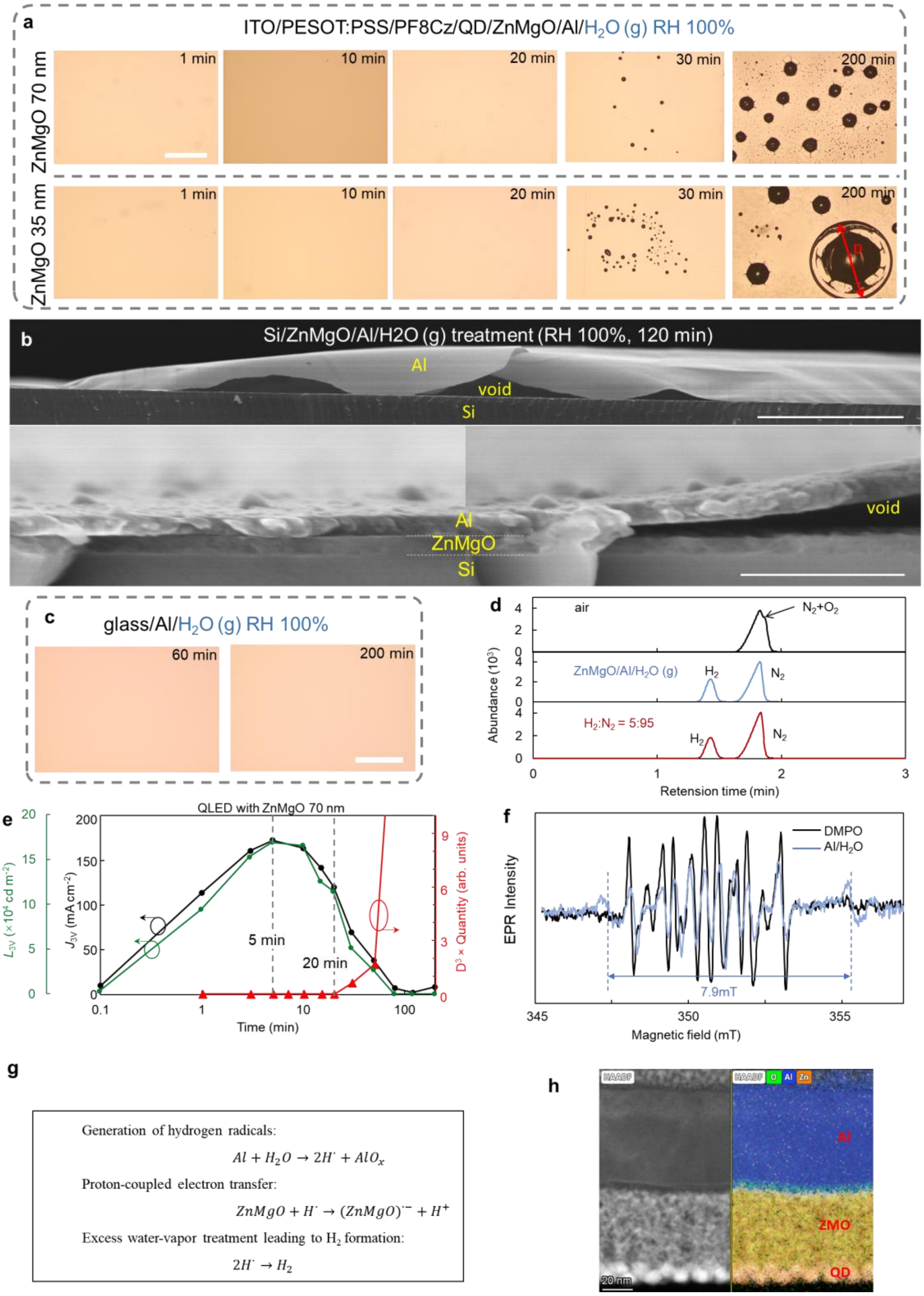

Supplementary Figure 2. QLED performance with excess water-vapor treatment (relative humidity (RH) 100%)

(a) Photographs of QLEDs after 100% RH water-vapor treatment with varied durations (upper: ZnMgO 70 nm, bottom: ZnMgO 35 nm, scale bar: 500 μm). Early appearance and large bubbles are reproducibly observed for the ZnMgO/Al film, consistent with formation of molecular hydrogen after the defect passivation and in situ reductive-doping. (b) Scanning electron microscopy images of the ZnMgO/Al film after excess water-vapor treatment. Voids seen in the images (top, low magnification, scale bar: 25

μm; bottom, high magnification, scale bar: 300 nm) are created by the hydrogen bubbles, which would disconnect the top electrode and the ZnMgO EIL and, in turn, destroy the QLEDs permanently. (c) Photographs of a glass/Al thin film after 100% RH water-vapor treatment with 60 and 200 minutes (scale bar: 500 μm). No appearance of bubbles indicates that formation of molecular hydrogen might occurs at the porous EIL and Al interface upon water-vapor treatment. (d) Gas chromatograms of air (top), hydrogen produced by reacting water vapor with ZnMgO/Al film in nitrogen (middle), and 5% hydrogen in nitrogen (bottom). (e) The current density, luminance at 3 V and total volume of bubbles are plotted against the water-vapor treatment duration. Observation of hydrogen bubbles is somewhat behind the defect passivation and in situ n-doping of the EIL. (f) The 298K EPR spectra of DMPO diluent (black line) and hydrogen radicals produced by reacting water vapor and Al, and captured by the DMPO diluent (blue line). The hyperfine splitting of 7.9 mT signifies the DMPO-captured hydrogen radicals. (g) Key reactions occur during water-vapor treatment on ZnMgO/Al. (h) A cross-sectional high-angle annular dark- field (HAADF) image and a color-coded elemental mapping image shows the accumulation of O-species at ZnMgO/Al interface. Given the roughness of the Al deposition on the nanoporous ZnMgO EIL, the AlO_x layer formed at the Al-ZnMgO interface by the water-vapor treatment is estimated to be < 1 nm and non-continuous, which should not significantly impede the electron conductivity of the EIL.

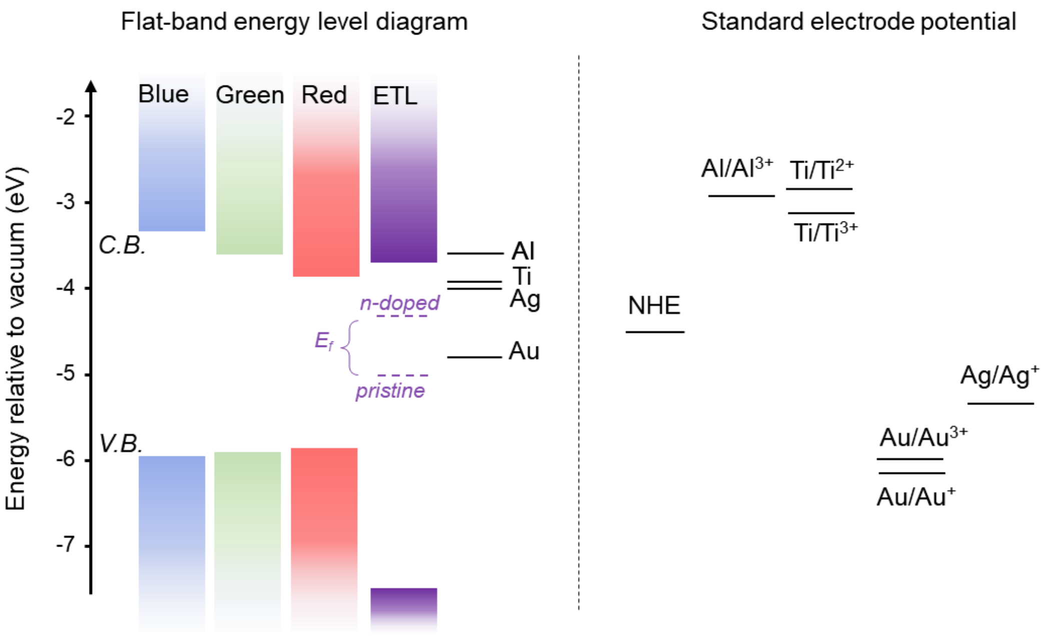

Supplementary Figure 3. Energy level alignment and electrochemical reactivity of cathode metal used in this work

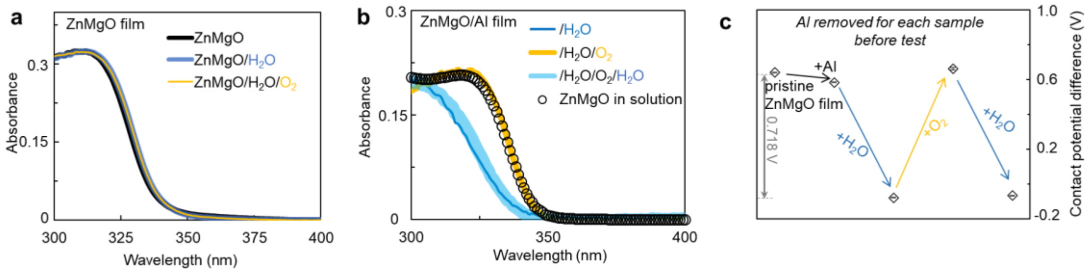

Supplementary Figure 4. The recovery of n-doping attributes of ZnMgO nanocrystals in the EIL via water-vapor treatment

(a) Absorption spectra of pristine ZnMgO film (without the Al electrode) following different treatments. (b) The absorption spectra for the ZnMgO/Al film following water-vapor treatment, oxygen treatment, and a subsequent second round of water-vapor treatment. For comparative purposes, the absorption spectra of ZnMgO in an ethanol solution are also included (marked with black circles). The general trend observed is consistent with that shown in Fig. 1c. Notably, the water-vapor treatment subsequent to the oxygen treatment appears to revert the film's properties to a status akin to that achieved after the initial water-vapor treatment. (c) The surface potential measurements of the ZnMgO/Al films are depicted after exposure to various atmospheric conditions, with all measurements performed after removal of the Al electrodes. Intriguingly, the sequence of water-vapor treatment following oxygen treatment effectively restores the n-type doping characteristics, mirroring the effects observed after the initial water treatment of the pristine ZnMgO film. In addition, the Fermi level of the oxygen-treated ZnMgO film is almost identical to that of the pristine ZnMgO film, which, along with the ultraviolet-visible absorption measurements in (b), implies that the defect states in the pristine ZnMgO are likely similar to those created by the oxygen treatment.

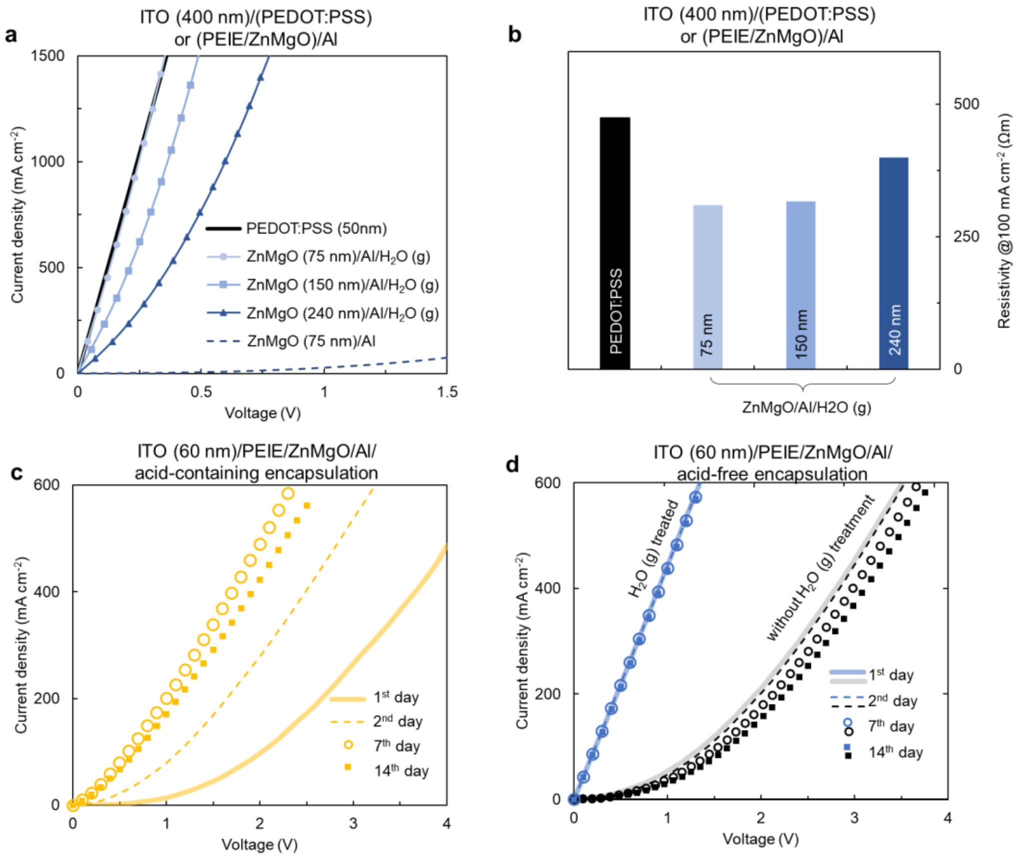

Supplementary Figure 5. Conductivity measurements of ZnMgO films

(a) The *J*-*V* characteristics of PEDOT:PSS film, pristine ZnMgO film and n-doped ZnMgO films with varying thicknesses. (b) The calculated conductivity of films in (a). (c) *J*-*V* measurements of ZnMgO/Al films with the acid-containing encapsulation, which were stored for different durations to present the stability of conductivity. (d) *J*-*V* measurements of ZnMgO/Al films with and without water-vapor treatment, encapsulated with acid-free resin.

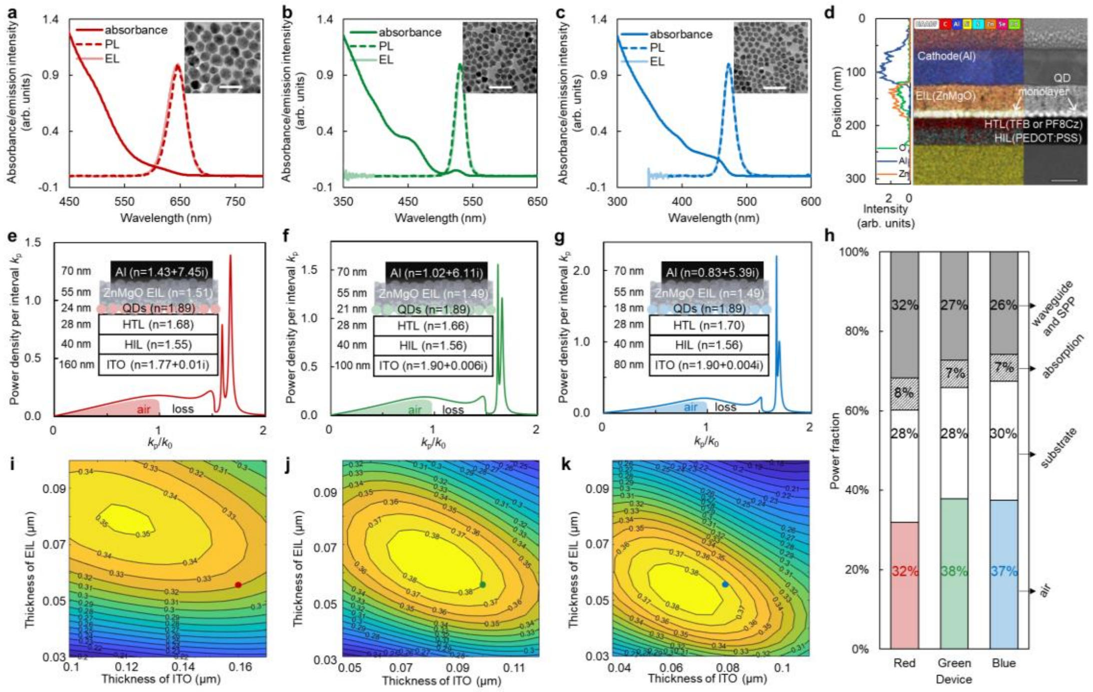

Supplementary Figure 6. Characterization of typical CdSe/$Cd_xZn_ySe_zS$/ZnS core/shell/shell QDs and QLEDs, along with optical modeling of QLEDs

(a-c) TEM images (insets, scale bar: 50 nm), ultraviolet-visible absorption, photoluminescence, and electroluminescence spectra of the red-emitting (644-nm) (a), green-emitting (532-nm) (b), and blue-emitting (474-nm) (c) QDs. (d) Position-dependent elemental profiles (left and middle) and a cross-sectional TEM image (right) of a green QLED. Scale bar: 50 nm. (e-g) Thicknesses and refractive indices of the multilayers (insets) and power density distributions as a function of in-plane wavevectors (k_p) for the red (e), green (f), and blue (g) QLEDs. k_0 represents the wavevector in vacuum. (h) Calculated power fractions of optical modes for the three devices. “air”, “substrate”, “absorption”, and “waveguide and SPP” correspond to the power factions out-coupled into air (i.e., light-extraction efficiency), trapped within the glass substrate—the part can be extracted using hemispherical lens, absorbed by the medium, and coupled into waveguide modes or surface plasmon polaritons, respectively. (i-k) Effect of ITO and ZnMgO thickness on the light extraction efficiency are shown for red (i), green (j) and blue (k) QLEDs. The corresponding thicknesses used in our devices is marked with colored dots.

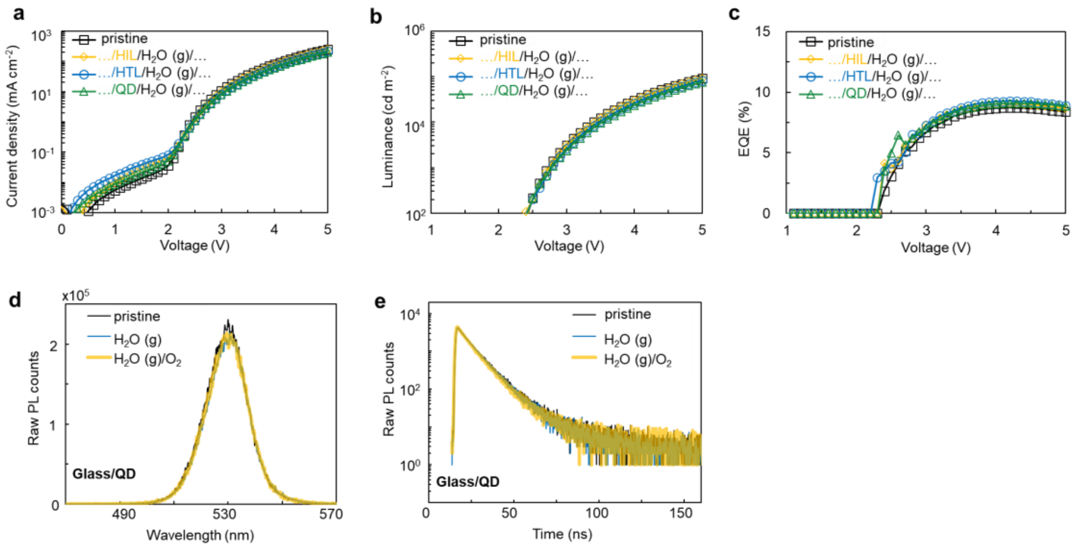

Supplementary Figure 7. Influence of water-vapor treatment on HIL, HTL and QD layer

(a) Current density, (b) luminance, and (c) EQE versus driving voltage for the green-emitting QLEDs that have undergone a water-vapor treatment at different stages of the manufacturing process. Following the water-vapor treatment, devices are completed using the standard procedure with the acid-free encapsulation. It is observed that there is minimal impact if the water treatment is applied to layers prior to the deposition of ZnMgO. (d) Steady-state, and (e) transient photoluminescence spectra of a QD film treated with different atmospheres.

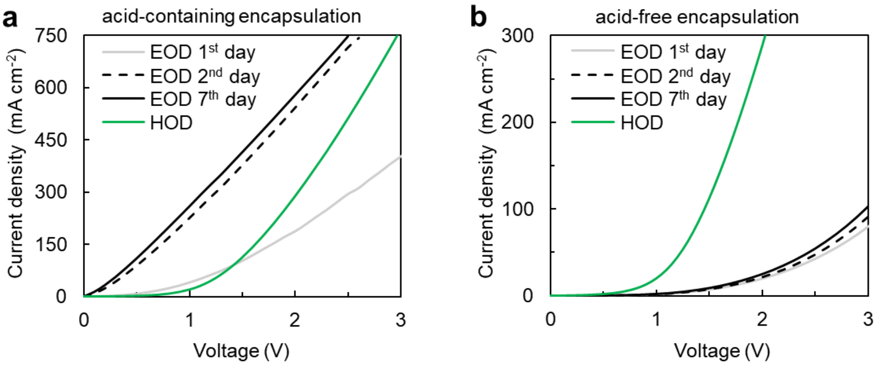

Supplementary Figure 8. *J-V* characteristics of single carrier devices

(a) With acid-containing encapsulation, the electron current is relatively low on the 1st day, which increases significantly on the 2nd day and saturates after 1 week's storage. (b) With acid-free encapsulation, the electron current of electron-only device (EOD, with structure ITO/PEIE/QD/EIL/Al) is stable against on-shelf storage, while largely lags behind hole current measured from a hole-only device (HOD, with structure ITO/HIL/HTL/QD/MoO_x/Au).

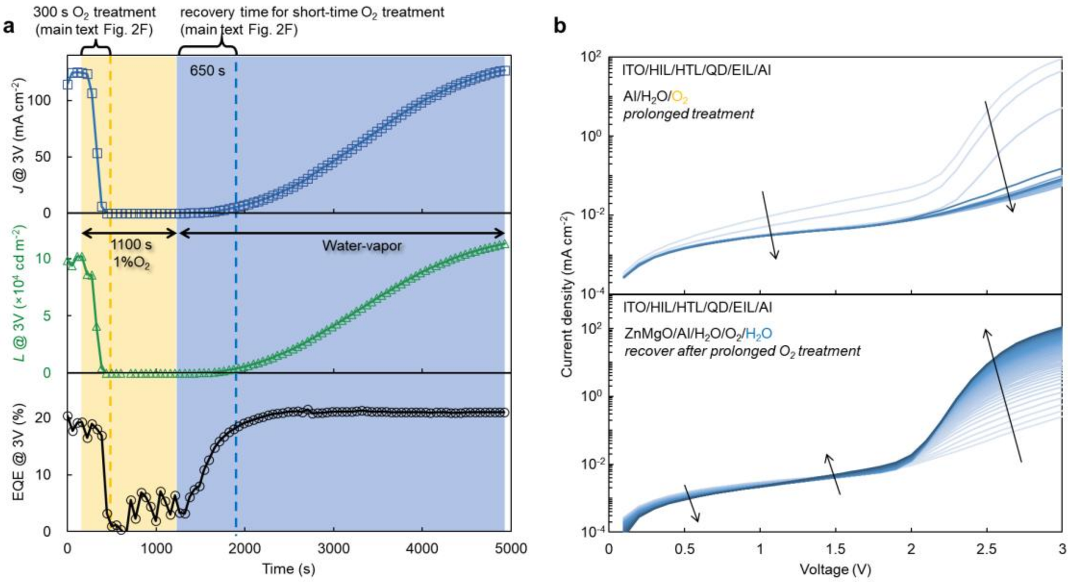

Supplementary Figure 9. Prolonged oxygen treatment slows recovery

(a) In situ monitoring of the current density (J_{3V}), luminance (L_{3V}) and EQE at 3.0 V over time for a device subjected to an extended oxygen treatment (1100 seconds compared to 300 seconds in Fig. 2f,g) followed by a full recovery through an extended water-vapor treatment. (b) The corresponding in situ current density (J) versus voltage curves for the prolonged oxygen treatment (top) and then the following water-vapor treatment (bottom). The device was pre-treated with water-vapor to achieve full recovery.

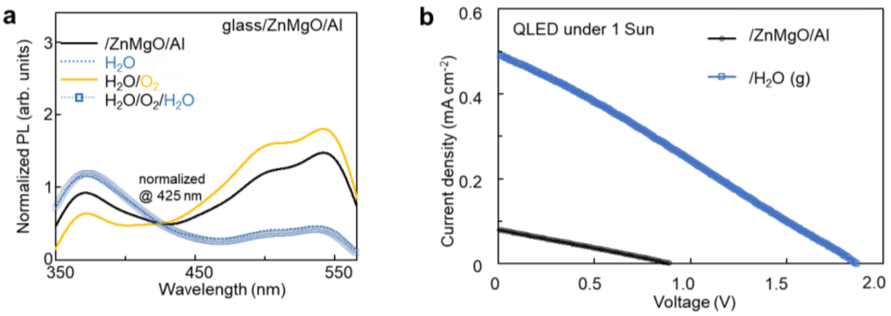

Supplementary Figure 10. PL spectra of a ZnMgO/Al film treated by different atmospheres and a QLED (532 nm) operating in photovoltaic mode

(a) The normalized PL spectra of the glass/ZnMgO/Al film treated with different atmospheres. (b) The water-vapor treatment leads to a notable increase of both the open-circuit voltage and the short-circuit current of the QLED, supporting reduction of the defect-related recombination centers by the water-vapor treatment of the ZnMgO EIL with the Al electrode. The steeper slope at the open-circuit voltage point indicates a lower series resistance contributed by the in situ n-doped ZnMgO EIL.

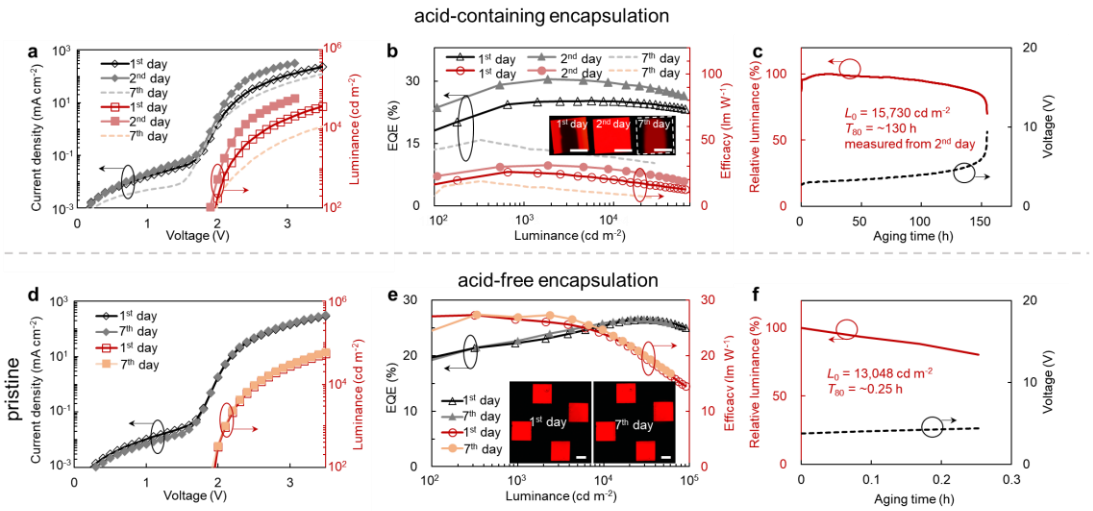

Supplementary Figure 11. Performance of red-emitting (644 nm) QLEDs

J-*V*-*L* characteristics (a), and electroluminescence photographs (insets, scale bar: 1 mm), EQEs and efficacies versus luminance (b) of a QLED with the acid-containing encapsulation (LOCTITE 3492) and stored for 1, 2 and 7 days. After storage for 1 day, the peak EQE, current density and luminance at 3.0 V of the device are significantly improved, accompanied by the luminance uniformity evolvement (gradually brightening from the edges to the center). However, negative aging effect occurs at the 7th day. (c) Relative luminance and voltages versus operational time of a red-emitting QLED after storage for two days, with an initial luminance of 15,730 cd m^{-2}, showing a large increase in the driving voltage and a sudden drop (jump) of luminance (voltage) at ~150 hours. (d-f) The same characterizations as in (a-c), but on the device with the acid-free encapsulation. After storage, the device exhibits negligible changes. In all insets, scale bar: 1 mm.

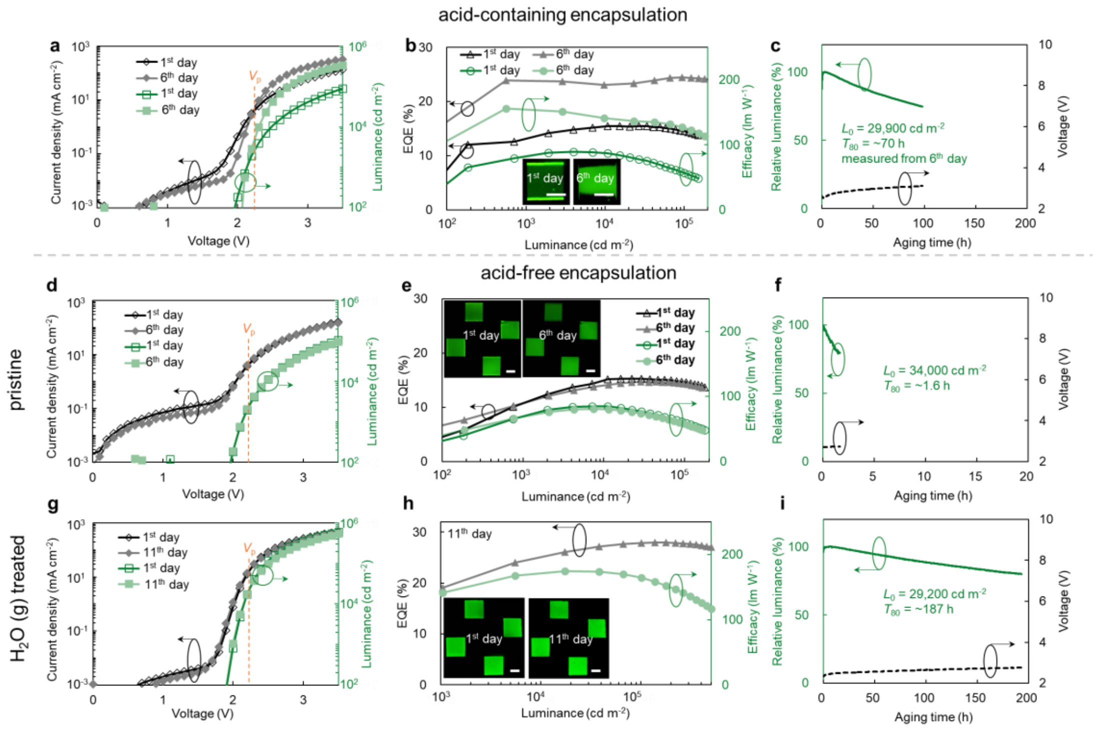

Supplementary Figure 12. Performance of green-emitting (556 nm) QLEDs

(a,b) *J-V-L* characteristics (a), electroluminescence photographs (insets, scale bar: 1 mm), EQEs and efficacies versus luminance (b) of a QLED with the acid-containing encapsulation and stored for 1 day and 6 days. After storage, the peak EQE, current density and luminance at 3.0 V of the device are significantly improved, accompanied by the luminance uniformity evolvement (gradually brightening from the edges to the center and darkened at the edges seen in photographs). (c) Relative luminance and voltages versus operational time, with an initial luminance of 29,900 cd m^{-2}, showing a large increase in the driving voltage. (d-f) The same characterizations as in (a-c), but on the device with the acid-free encapsulation. After storage, the device exhibits negligible changes. (g-i) The same characterizations as in (a-c), but on the typical QLED with water-vapor treatment prior to the acid-free encapsulation. Negligible change in *J-V-L* is observed after storage for 11 days. The luminance at V_p is 9-fold higher than that of a QLED without water-vapor treatment (d). The T_{80} lifetime for the device treated with water-vapor is ~100 times longer than that without treatment (f). In all insets, scale bar: 1 mm.

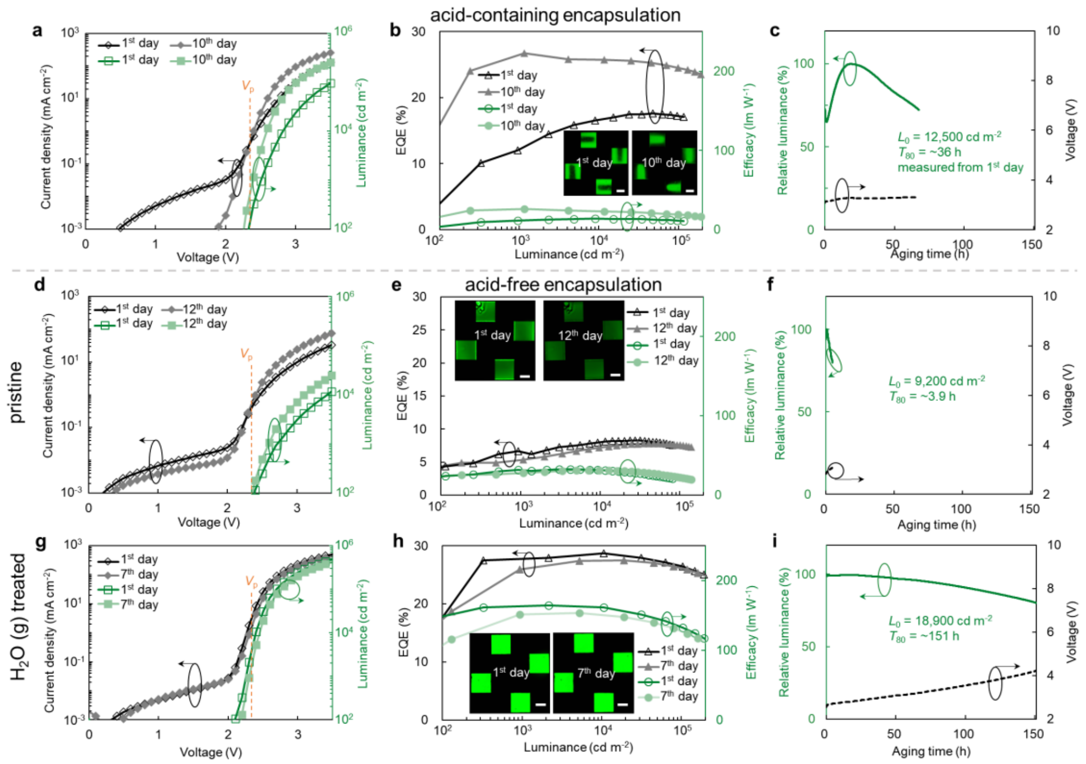

Supplementary Figure 13. Performance of green-emitting (532 nm) QLEDs

(a,b) *J-V-L* characteristics (a), electroluminescence photographs (insets, scale bar: 1 mm), EQEs and efficacies versus luminance (b) of a QLED with the acid-containing encapsulation and stored for 1 day and 10 days. After storage, the peak EQE, current density and luminance at 3.0 V of the device are significantly improved, accompanied by the luminance uniformity evolvement (gradually brightening from the edges to the center and darkened at the edges). (c) Relative luminance and voltages versus operational time, with an initial luminance of 12,500 cd m^{-2}, showing a large increase in the driving voltage. (d-f) The same characterizations as in (a-c), but on the device with the acid-free encapsulation. After storage, the device exhibits negligible changes. (g-i) The same characterizations as in (a-c), but on the typical QLED with water-vapor treatment prior to the acid-free encapsulation. Negligible change in *J-V-L* is observed after storage for 7 days. The T_{80} lifetime for the device treated with water-vapor is ~38 times longer than that without treatment (f). In all insets, scale bar: 1 mm.

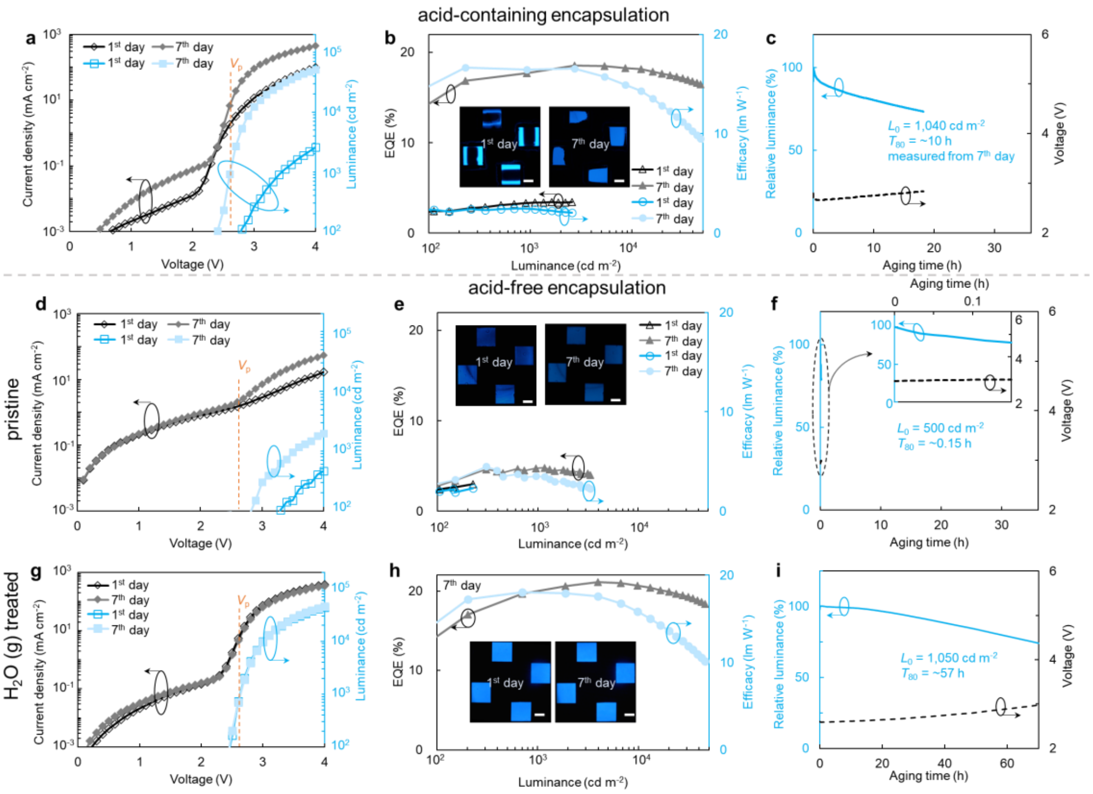

Supplementary Figure 14. Performance of blue-emitting (474 nm) QLEDs

(a,b) *J*-*V*-*L* characteristics (a), electroluminescence photographs (insets, scale bar: 1 mm), EQEs and efficacies versus luminance (b) of a QLED with the acid-containing encapsulation and stored for 1 day and 7 days. After storage, the peak EQE, current density and luminance at 3.0 V of the device are significantly improved, accompanied by the luminance uniformity evolvement. (c) Relative luminance and voltages versus operational time, with an initial luminance of 1,040 cd m^{-2}. (d-f) The same characterizations as in (a-c), but on the device with the acid-free encapsulation. After storage, the device exhibits a slight increase in current density, brightness, EQE and efficacy probably due to unintentional exposure to moisture during device fabrication. (g-i) The same characterizations as in (a-c), but on the typical QLED with water-vapor treatment prior to the acid-free encapsulation. Negligible change is observed *J*-*V*-*L* after storage for 7 days. The luminance at 3.0 V is 50-fold higher than that of a QLED without the water-vapor treatment (d). The T_{80} lifetime for the device with the water-vapor treatment (@1,050 cd m^{-2}) is 380 times longer than that without the water treatment (@500 cd m^{-2}) in (f). The most pronounced improvements by the water-vapor treatment are observed for the blue-QLEDs. In all insets, scale bar: 1 mm.

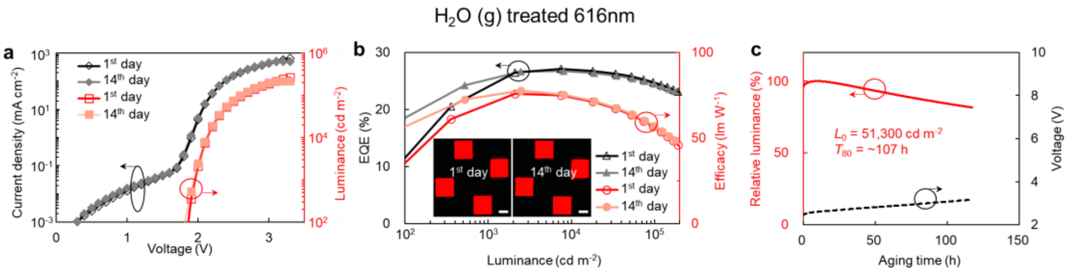

Supplementary Figure 15. Performance of orange-red-emitting (616 nm) QLEDs *J*-*V*-*L* characteristics (a), and electroluminescence photographs (insets, scale bar: 1 mm), EQEs and efficacies versus luminance (b) of a QLED stored for 1 day and 14 days. (c) Relative luminance and voltages versus operational time, with an initial luminance of 51,300 cd m^{-2}. In all insets, scale bar: 1 mm.

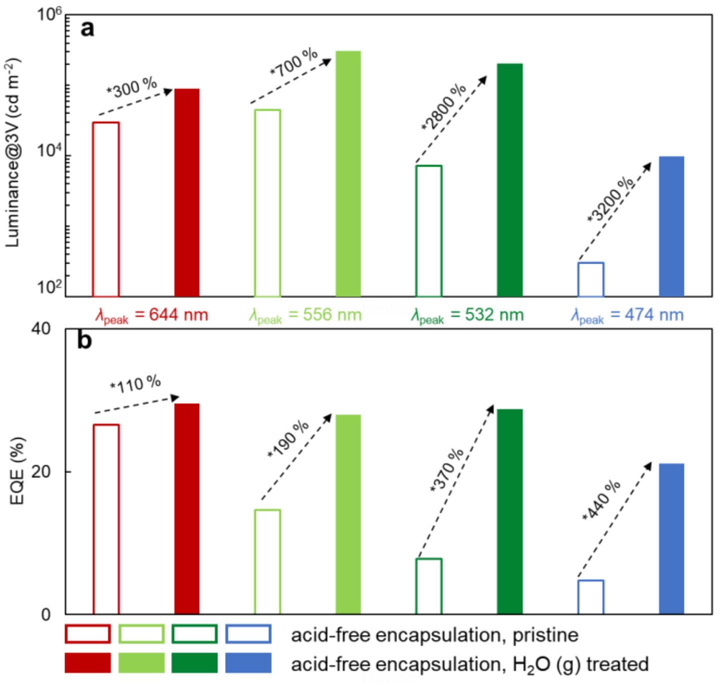

Supplementary Figure 16. Water-vapor treatment boosts luminance and EQE more at shorter wavelengths

Comparison of the effect of water-vapor treatment on QLEDs emitting at different wavelengths, in terms of luminance (a) and EQE (b). An increased enhancement in both luminance and EQE can be observed when the wavelength becomes shorter.

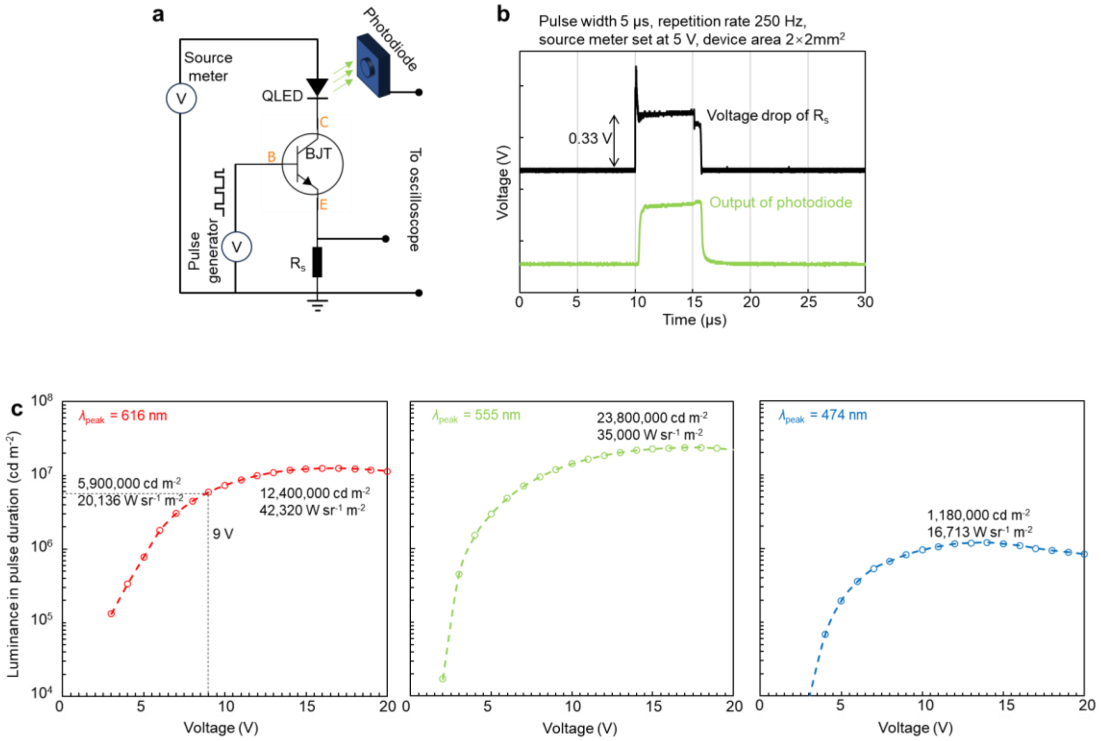

Supplementary Figure 17. Characterization on QLEDs under electrical pulse excitation

(a) Setup used for electrical pulse excitation. (b) Voltage-drop across the sampling resistance and the response of a photodiode that captures the optical signal emitted by the QLED, demonstrating a 5-μs electrical-excitation and light-emitting pulse. (c) The corrected (see Methods) luminance-voltage characteristics for red, green and blue QLEDs. All devices tested in (c) have an active area of 0.02 mm^2.

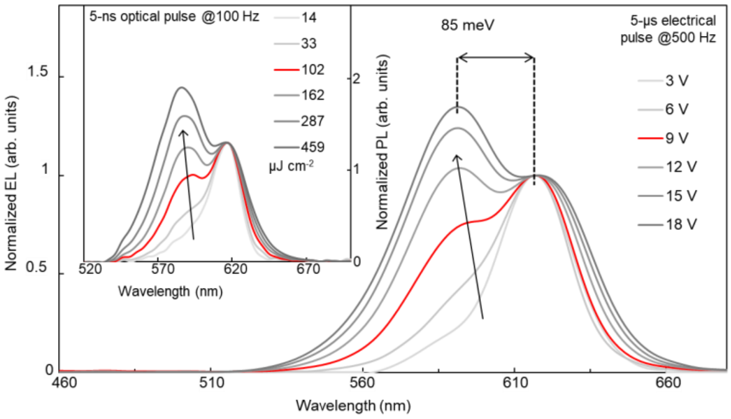

Supplementary Figure 18. Voltage-dependent EL spectra of a red QLED under pulse excitation

Voltage-dependent electroluminescence spectra of a red-emitting (616-nm) QLED excited by electrical pulses, with an emissive area of 0.02 mm^2. Inset: Photoluminescence spectra under 5-nanoseconds pulsed laser excitation with varied power densities (signal collected from out-of-plane direction).

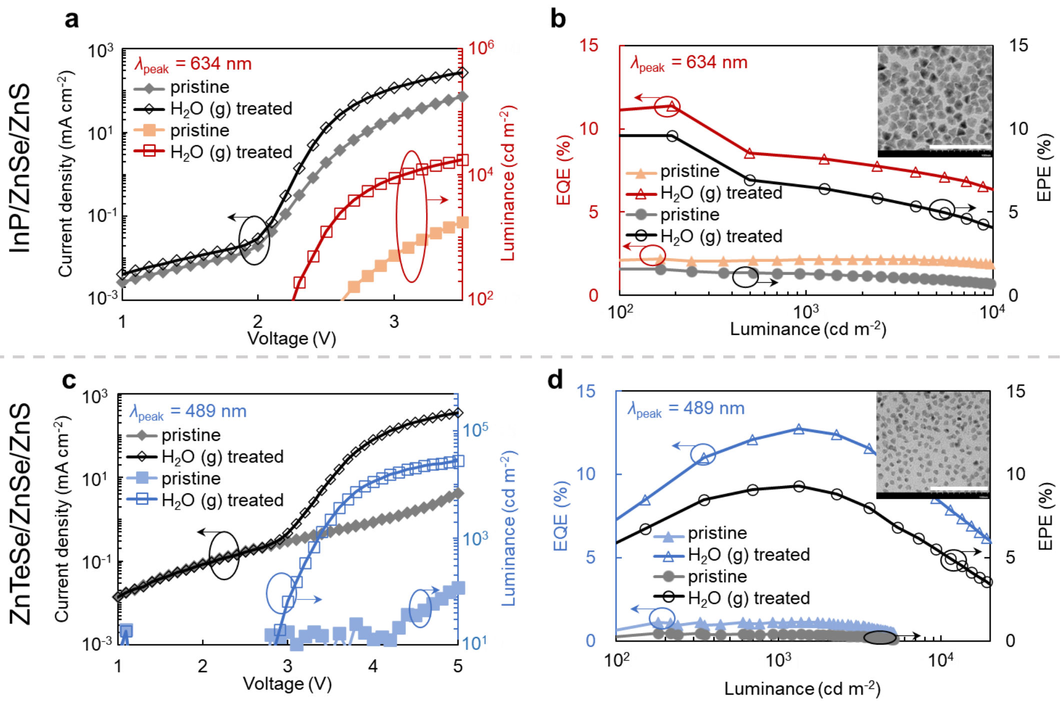

Supplementary Figure 19. The effect of water-vapor treatment on InP-based (634 nm) and ZnTeSe-based (489 nm) QLEDs

(a,b) *J*-*V*-*L* characteristics (a), EQEs and EPE versus luminance (b) of an InP-based QLED with the acid-free encapsulation (pristine) and treated by water vapor (H_2O (g) treated). Inset, the TEM images of the QDs used (scale bar: 100nm). (c,d) The same characterizations as in (a,b), but on a ZnTeSe-based QLED.

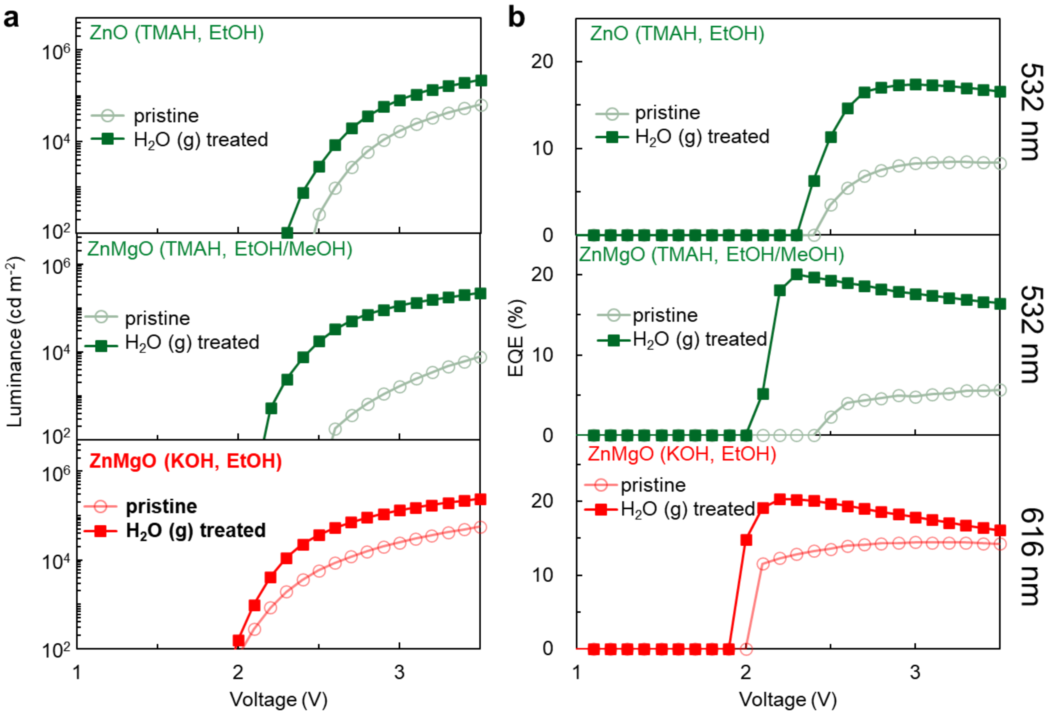

Supplementary Figure 20. Performance of the green-emitting and orange-red-emitting (532 and 616 nm) QLEDs with different electron injection layers

(a,b) Luminance (a), and EQE (b) curves of the devices with a pristine and a water-treated electron injection layer, based on ZnO (synthesized with TMAH in ethanol), ZnMgO (synthesized with TMAH in ethanol/methanol mixture), and ZnMgO (synthesized with KOH in ethanol). See Methods for details. QLEDs treated with water vapor exhibit marked enhancements in current density, luminance, and EQE compared to their untreated counterparts.

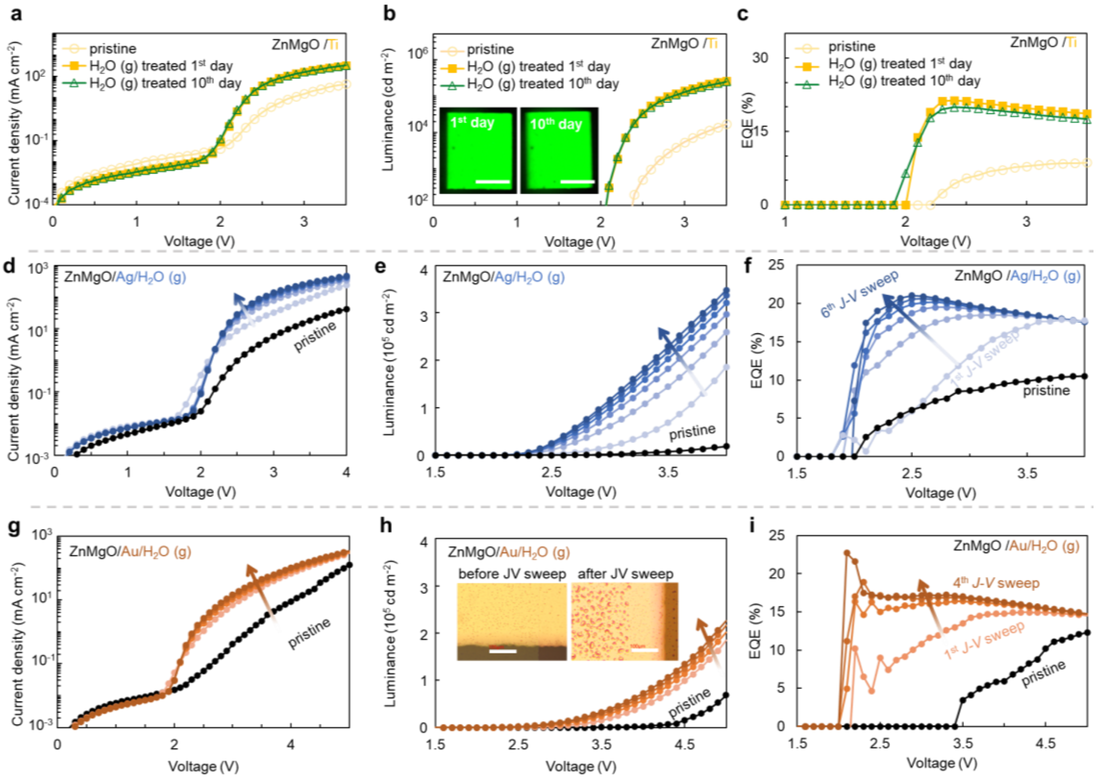

Supplementary Figure 21. Performance of the green-emitting (532 nm) QLEDs with different top electrodes

(a-c) Current density (a), luminance and photographs of electroluminescence (inset, scale bar: 1 mm) (b), and EQE (c) curves for the QLEDs with a titanium electrode. Both pristine device and the one with a water-vapor treatment after deposition of the top electrode (H_2O (g) treated) are shown (with varying shelf-aging time as marked). (d-f) Current density (d), luminance (e), and EQE (f) versus voltage of a device with a silver electrode before the water treatment (pristine) and in six sequential cyclic sweeps between 0 V to 4 V after the water treatment (blue). (g-i) Current density (g), luminance (h), and EQE (i) versus voltage of a device with a gold electrode before the water treatment (pristine) and in four sequential cyclic sweeps between 0 V to 5 V after the water treatment. Inset of (h) show the photograph of QLED with over-dose water-vapor treatment before and after *J-V* measurement (scale bar: 200 μm). Small bubbles emerge due to the hydrogen gas generated from the electrochemical reduction of adsorbed H_2O, occurring at the gold cathode.

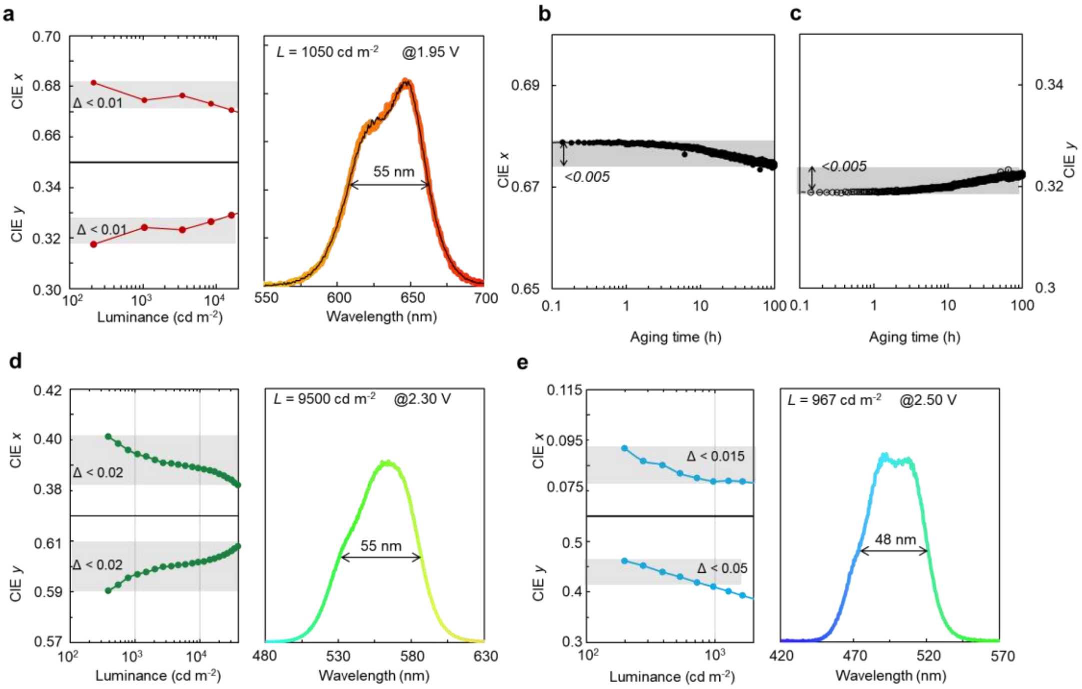

f

Color	ϕ_L(lm)	ϕ_L ratio	S (m^2)	L (cd m^{-2})	P_E ratio	V_p (V)	η_L (lm W^{-1})	EPE (%)
Red-orange	1160	29%	0.24	1539	46.6% * (40.2%)**	2.017	312	55
Yellow-green	2280	57%	0.24	3025	28.1%* (29.0%)**	2.268	596	46
Green-blue	560	14%	0.24	743	25.3% * (30.8%)**	2.570	159	32

ϕ_L: luminous flux, L: luminance, S: emitting area, η_L: luminous efficacy, P_E: electrical power
*external power efficiency (EPE) = 100% is assumed
**certificated EPEs (last column) in the spectral range are taken into consideration

Supplementary Figure 22. Color stability and luminous efficacy of red, green, and blue βQLEDs

(a) CIE-x and y values of the red βQLED in Fig. 5b as a function of luminance. (b,c) The color drift of a red βQLED during 100 hours continuous operation under a constant bias of 1.90 V. (d,e) CIE-x and y values of the green and blue βQLED as a function of luminance and representative spectra acquired at bandgap voltage. The green βQLED incorporate QDs emitting at 532, 551 and 571 nm and the blue βQLED utilize QDs emitting at 471, 489 and 510 nm. (f) Key parameters used in luminous efficacy estimation of the proposed white-light QLED.

No.	T2403WT8888-001923-Y
Total page	7

TEST REPORT

Partial copying without authorization is prohibited

Product Name :	red- green- and blue-QLEDs with hemispherical out-coupling lens
Type and Specification :	/
Test Category :	Entrusted Test
Factory :	/
Client:	Zhejiang University

China Electronic Product Reliability And Environmental Testing Research Institute

CEPREI China CEPREI Laboratory

668420

Page 2 of 7 Report ref. No. T2403WT8888-001923-Y

TEST REPORT

Report reference No ....:	T2403WT8888-001923-Y
Total number of pages........................:	7
Test item description ....:	red- green- and blue-QLEDs with hemispherical out-coupling lens
Trademark ....:	/
Model and/or type reference ....:	/
Factory's Name ....:	/
Address ....:	/
Client's Name ....:	Zhejiang University
Address ....:	866 Yuhangtang Rd, Hangzhou 310058, P.R. China
Testing Laboratory Name ....:	China CEPREI Laboratory/ China Electronic Product Reliability and Environmental Testing Research Institute
Address ....:	No.78, West of Zhucun Road, Zhucun Street, Zengcheng District, Guangzhou, Guangdong, China
Testing location ....:	No.78, West of Zhucun Road, Zhucun Street, Zengcheng District, Guangzhou, Guangdong, China
Test specification	
Standard........................:	Request from the client
Test category ....:	Entrusted Test
Number of test item ....:	3pcs
Date of receipt of test item ....:	2024-02-28
Date(s) of performance of test..........:	2024-03-25
Ambient Condition........................:	25.0℃~25.2℃, 53%RH~58%RH
Test Instruments and Equipment......:	See Equipment List of This Report.

Summary of Testing and Conclusions

No judgment，details see page 6.

Tested by (printed name and signature) ....:	Zheng Guanxiong（郑冠雄）
Reviewed by (printed name and signature) ....:	Wang Zhen（王振）
Approved by (printed name and signature) ....:	Liu Ju（刘菊）
Date of issue........................:	2024 年 3 月 29 日

CEPREI-AQ/WT003-202005

Page 3 of 7 Report ref. No. T2403WT8888-001923-Y

Test case verdicts	
Test case does not apply to the test object....................:	N/A
Test item does meet the requirement(Pass) ....:	P
Test item does not meet the requirement(Fail)............:	F

General remarks

1. Report without "specific stamp" of inspection organization or the authority will be regarded as invalid.
2. Duplicated report without original "specific stamp" of inspection organization or the authority will be regarded as invalid.
3. Report without the signatures of Tester, Reviewer or Approval will be regarded as invalid.
4. Test report if altered will be regarded as invalid.
5. Any dispute about the report must be submitted to inspection organization within 15 days upon report received, it will be rejected if out of the period.
6. Generally, the entrusted test only responsible for the samples.

Page 4 of 7 Report ref. No. T2403WT8888-001923-Y

General product information:

1. Description of the sample

No.	Test voltage
#39	2. 2V
	2. 4V
	3. 0V
#255	2. 6V
	3. 0V
#23	2. 8V
	3. 0V
	3. 2V

2. Testing items、Inspection methods and judgments

No.	Testing items	Inspection methods and judgments
1	current	The client requires the use of a Multimeter to test current. Judgment requirements:/
2	Luminous flux	The client requires the use of a Integrating sphere to test Luminous flux. Judgment requirements:/
3	Radiant flux	The client requires the use of a Integrating sphere to test Radiant flux. Judgment requirements:/
4	Peak wavelength	The client requires the use of a Integrating sphere to test Peak wavelength. Judgment requirements:/
5	Efficacy	The client requests to use an integrating sphere to test Luminous flux and then calculate Efficacy using the formula. Judgment requirements:/
6	EPE(external power efficiency)	The client requests to use an integrating sphere to test Radiant flux and then calculate EPE(external power efficiency) using the formula. Judgment requirements:/

3. The client requires measurement to be conducted after lighting up the light source for 1 minute.

Testing Laboratory Contact Info:

China CEPREI Laboratory/ China Electronic Product Reliability and Environmental Testing Research Institute

Address: No.78, West of Zhucun Road, Zhucun Street, Zengcheng District, Guangzhou, Guangdong, China

Postcode: 511370

Business Contact: +86-20-85131039,market@ceprei.biz

FAX: +86-20-85131313

Enquiry：+86-20-85131123,info@ceprei.biz

Complaint：+86-20-85131208, +86-20-87236881

CEPREI-AQ/WT002-202005

Page 5 of 7 Report ref. No. T2403WT8888-001923-Y

Photo of test

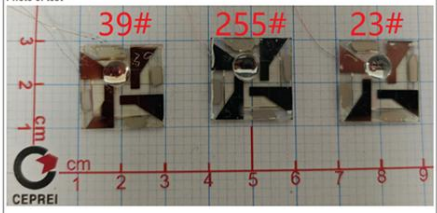

Page 6 of 7 Report ref. No. T2403WT8888-001923-Y

Detection result

No.	input voltage (V)	current (mA)	Luminous flux (lm)	Radiant flux (mW)	Peak wavelength (nm)	Efficacy (lm/W)	EPE (external power efficiency) (%)
#39	2. 2V	0. 91	0. 12484	1. 1096	648	62. 36	55. 42
	2. 4V	2. 64	0. 36156	3. 1166	647	57. 06	49. 19
	3. 0V	13. 56	1. 7810	14. 335	646	43. 78	35. 24
#255	2. 6V	0. 55	0. 38343	0. 65965	532	268. 13	46. 13
	3. 0V	4. 65	3. 0180	5. 1847	532	216. 34	37. 17
#23	2. 8V	0. 94	0. 074933	0. 84332	472	28. 47	32. 04
	3. 0V	2. 15	0. 15993	1. 8570	470	24. 80	28. 79
	3. 2V	3. 51	0. 24037	2. 8306	470	21. 40	25. 20

Remarks: The calculation formula is provided by the client
Efficacy= Luminous flux/（voltage×current）
EPE(external power efficiency)= Radiant flux/（voltage×current）×100%

Page 7 of 7 Report ref. No. T2403WT8888-001923-Y

Test Instruments and Equipment

No.	Equipment	Model	Series No.	Calibration date to
1	Digital temperature and humidity meter	DL336001	WSDJ202301	2023. 12. 21-2024. 12. 20
2	LED photoelectric analysis and measurement system	HAAS-2000	AQDC-001	2024. 01. 05-2025. 01. 04
3	Digital source table	2400	4321702	2023. 10. 11-2024. 10. 10
4	Multimeter	34450A	MY55050003	2023. 12. 19-2024. 12. 18

Supplementary Figure 23. Performance of devices certificated by China Electronic Product Reliability and Environmental Testing Research Institute

Color	λ_{peak} (nm)	EQE_{max} (%)	$L@V_p$ (cd m^{-2})	L_{3V} (cd m^{-2})	L_{max} (cd m^{-2})	Normalized lifetime* (h)	Encapsulation **	Reference
Red	640	20.4	10	1,100	42,000	T_{80}@30k nit=7.3	--	7
	645	22.9	1,120	38,000	--	T_{95}@30k nit=145	--	8
	640	35.6***	500	57,000	--	T_{95}@30k nit=76	--	9
	644	28.9	2,900	94,000	673,000	T_{95}@30k nit=111<br>T_{80}@30k nit=450	Acid-free	This work
Orange-red	625	13.4	620	8,000	--	T_{95}@51k nit=3	--	10
	620	20	178	7,000	--	T_{80}@51k nit=52	Acid-free	1
	600	21.6	40	5,000	356,000	T_{80}@51k nit=16.4	--	11
	620	27.0	2,800	191,000	1,250,000	T_{95}@51k=41<br>T_{80}@51k=107	Acid-free	This work
Yellow-green	550	15	3,119	50,534	--	T_{95}@ 29k nit=29	--	8
	540	1.8	41	2,300	68,000		Acid-free	12
	556	29.5	24,700	359,000	3,100,000	T_{95}@29k nit=37<br>T_{80}@29k nit=187	Acid-free	This work
Green	537	28.7	100	40,000	--	T_{95}@18.9k nit=26	--	13
	530	22.9	40	2,200	614,000	T_{80}@18.9k nit=52	--	11
	532	28.3	4,100	231,000	2,480,000	T_{95}@18.9k nit=72<br>T_{80}@18.9k nit=151	Acid-free	This work
Blue	479	21.9	10	900	--	T_{95}@1.05k nit=52	--	13
	480	8.1	40	50	63,000	T_{80}@1.05k nit=28	--	11
	482	10	262	4,235	--	T_{95}@1.05k nit=57	--	8
	467	12.6	12	159	--	T_{80}@1.1k nit=7.5	--	14
	482	20.4	--	0.1	--	T_{95}@1k nit=227	--	15
	473	21.1	920	9,760	207,000	T_{95}@1.05k nit=25<br>T_{80}@1.05k nit=57	Acid-free	This work

*Calculated at specific luminance using the reported acceleration factor in each literature.

**'--' means the resin used is not mentioned in literature.

***in-plane dipole optimized

Supplementary Table 1. Comparisons of our champion devices with top-performing QLEDs reported in literature[1,7-15]

Supplementary References

1. Chen, D. *et al.* Shelf-Stable Quantum-Dot Light-Emitting Diodes with High Operational Performance. *Adv. Mater.* **32**, 2006178, (2020).
2. Jia, S. *et al.* Optimizing ZnO–Quantum Dot Interface with Thiol as Ligand Modification for High-Performance Quantum Dot Light-Emitting Diodes. *Small* **20**, 2307298, (2024).
3. Chen, M. *et al.* Highly Stable SnO2-Based Quantum-Dot Light-Emitting Diodes with the Conventional Device Structure. *ACS Nano* **16**, 9631-9639, (2022).
4. Chen, Z., Qin, Z., Su, S. & Chen, S. The influence of H_2O and O_2 on the optoelectronic properties of inverted quantum-dot light-emitting diodes. *Nano Res.* **14**, 4140-4145, (2021).
5. Pu, C. *et al.* Electrochemically-stable ligands bridge the photoluminescence-electroluminescence gap of quantum dots. *Nat. Commun.* **11**, 937, (2020).
6. Kim, C. *et al.* Achieving Selective and Efficient Electrocatalytic Activity for CO2 Reduction Using Immobilized Silver Nanoparticles. *J. Am. Chem. Soc.* **137**, 13844-13850, (2015).
7. Dai, X. *et al.* Solution-processed, high-performance light-emitting diodes based on quantum dots. *Nature* **515**, 96-99, (2014).
8. Gao, Y. *et al.* Minimizing heat generation in quantum dot light-emitting diodes by increasing quasi-Fermi-level splitting. *Nat. Nanotechnol.* **18**, 1168-1174, (2023).
9. Xu, H. *et al.* Dipole–dipole-interaction-assisted self-assembly of quantum dots for highly efficient light-emitting diodes. *Nat. Photon.* **18**, 186-191, (2024).
10. Lin, J. *et al.* High-Performance Quantum-Dot Light-Emitting Diodes Using NiOx Hole-Injection Layers with a High and Stable Work Function. *Adv. Funct. Mater.* **30**, 1907265, (2020).
11. Shen, H. *et al.* Visible quantum dot light-emitting diodes with simultaneous high brightness and efficiency. *Nat. Photon.* **13**, 192-197, (2019).
12. Qian, L., Zheng, Y., Xue, J. & Holloway, P. H. Stable and efficient quantum-dot light-emitting diodes based on solution-processed multilayer structures. *Nat. Photon.* **5**, 543-548, (2011).
13. Deng, Y. *et al.* Solution-processed green and blue quantum-dot light-emitting diodes with eliminated charge leakage. *Nat. Photon.* **16**, 505-511, (2022).
14. Chen, S. *et al.* On the degradation mechanisms of quantum-dot light-emitting diodes. *Nat. Commun.* **10**, 765, (2019).
15. Chen, X. *et al.* Blue light-emitting diodes based on colloidal quantum dots with reduced surface-bulk coupling. *Nat. Commun.* **14**, 284, (2023).
